# Autophagy Controls Nrf2-Mediated Dichotomy in Pressure Overloaded Hearts

**DOI:** 10.3389/fphys.2021.673145

**Published:** 2021-05-13

**Authors:** Weiwei Wu, Qingyun Qin, Yan Ding, Huimei Zang, Dong-Sheng Li, Mitzi Nagarkatti, Prakash Nagarkatti, Wenjuan Wang, Xuejun Wang, Taixing Cui

**Affiliations:** ^1^Department of Cell Biology and Anatomy, School of Medicine, University of South Carolina, Columbia, SC, United States; ^2^Department of Cardiology, Tianjing First Central Hospital, Tianjing, China; ^3^Hubei Key Laboratory of Embryonic Stem Cell Research, Taihe Hospital, Hubei University of Medicine, Shiyan, Hubei, China; ^4^Department of Pathology, Microbiology and Immunology, School of Medicine, University of South Carolina, Columbia, SC, United States; ^5^Vascular Biology Center and Department of Pharmacology and Toxicology, Medical College of Georgia, Augusta University, Augusta, GA, United States; ^6^Division of Basic Biomedical Sciences, Sanford School of Medicine, University of South Dakota, Vermillion, SD, United States

**Keywords:** Nrf2, autophagy, ERK, cardiac dysfunction, pressure overload

## Abstract

Burgeoning evidence has indicated that normal autophagy is required for nuclear factor erythroid 2-related factor (Nrf2)-mediated cardiac protection whereas autophagy inhibition turns on Nrf2-mediated myocardial damage and dysfunction in a setting of pressure overload (PO). However, such a concept remains to be fully established by a careful genetic interrogation *in vivo*. This study was designed to validate the hypothesis using a mouse model of PO-induced cardiomyopathy and heart failure, in which cardiac autophagy and/or Nrf2 activity are genetically inhibited. Myocardial autophagy inhibition was induced by cardiomyocyte-restricted (CR) knockout (KO) of autophagy related (Atg) 5 (CR-Atg5KO) in adult mice. CR-Atg5KO impaired cardiac adaptations while exacerbating cardiac maladaptive responses in the setting of PO. Notably, it also turned off Nrf2-mediated defense while switching on Nrf2-operated tissue damage in PO hearts. In addition, cardiac autophagy inhibition selectively inactivated extracellular signal regulated kinase (ERK), which coincided with increased nuclear accumulation of Nrf2 and decreased nuclear translocation of activated ERK in cardiomyocytes in PO hearts. Mechanistic investigation revealed that autophagy is required for the activation of ERK, which suppresses Nrf2-driven expression of angiotensinogen in cardiomyocytes. Taken together, these results provide direct evidence consolidating the notion that normal autophagy enables Nrf2-operated adaptation while switching off Nrf2-mediated maladaptive responses in PO hearts partly through suppressing Nrf2-driven angiotensinogen expression in cardiomyocytes.

## Introduction

Autophagy is an evolutionarily conserved pathway that targets cytoplasmic contents to the lysosome for degradation (Wang and Hill, 2015; Wang and Cui, 2017; Sciarretta et al., 2018). Based on how the target is delivered into lysosomes for final degradation, autophagy in mammals has been classified into three types: (i) macroautophagy, (ii) microautophagy, and (iii) chaperone-mediated autophagy (CMA). The macroautophagy (thereafter referred to as autophagy) is the best characterized. Autophagy serves as a house keeping process to maintain cardiac integrity and function under baseline conditions. Though, the role of autophagy in stressed hearts remains controversial. Depending on the nature of stress and timing of assessment, activation of cardiac autophagy has been proposed to be either adaptive or maladaptive (Wang and Hill, 2015; Wang and Cui, 2017; Sciarretta et al., 2018). A biphasic modality of cardiac autophagy regulation in obesity has been proposed (Zhang et al., 2018). At an early stage of obesity, cardiac autophagy may be increased as an adaptive response; however, at a later stage of obesity, cardiac autophagy is impaired, which is characterized by increased initiation of autophagy and suppressed degradation of autophagosomes (Zhang et al., 2018). Importantly, most of the recent studies have shown that autophagy activation is likely an adaptive response in the hearts after pressure overload (PO hearts) (Wang and Cui, 2017; Sciarretta et al., 2018). In this regard, we lately demonstrated that PO-induced cardiomyopathy and heart failure are dramatically ameliorated by enhancing myocardial autophagy via cardiomyocyte-restricted (CR)-autophagy related 7 (Atg7) transgenic overexpression (Qi et al., 2020), providing critical evidence to support adaptive nature of autophagy activation in PO hearts. Nevertheless, the precise role of autophagy in stressed hearts remains to be investigated and it may not be fully understood until the signaling network of cardiac autophagy is completely mapped.

Nuclear factor-erythroid factor 2-related factor 2 (Nrf2), a transcription factor, controls the basal and inducible expression of several hundred genes that can be grouped into several categories with different functions including antioxidant defense, detoxification, inflammatory responses, gene transcription, transporters, protein degradation, and metabolism (Cui et al., 2016; Zang et al., 2020a). Thus, the functions of Nrf2 spread rather broadly from antioxidant defense to protein quality control and metabolism regulation. Historically, Nrf2 has been considered as a master regulator of antioxidant defense, thereby providing protection against diverse cardiomyopathies associated with oxidative stress (Li et al., 2009a). However, this notion is challenged by the emerging evidence which revealed a mediator role of Nrf2 in the progression of cardiomyopathies associated with various pathological settings including proteotoxicity associated with aging, myocardial ischemia-reperfusion injury, pressure overload, and type 1 diabetes (Zang et al., 2020a,b). Although the precise mechanisms underlying Nrf2-mediated dichotomy in the heart are poorly understood, we observed that activation of Nrf2 is protective (Li et al., 2009a) when autophagy is intact in PO hearts, whereas it become detrimental to PO hearts when cardiac autophagy is impaired (Qin et al., 2016). In addition, we found that autophagy inhibition via CR-Atg5KO activates Nrf2-driven expression of angiotensinogen in cardiomyocytes, presumably creating a pathological signaling axis in which autophagy inhibition activates Nrf2-driven angiotensinogen expression, which in turn promotes angiotensin II production and subsequent activation of angiotensin II receptor type 1 signaling, thereby contributing to cardiac pathological remodeling and dysfunction (Qin et al., 2016). These results indicate a unique role of autophagy in controlling Nrf2-mediated dichotomy in PO hearts, i.e., normal autophagy is required for Nrf2-mediated cardiac protection whereas autophagy inhibition turns on Nrf2-mediated myocardial damage and dysfunction. Yet, such a concept remains to be fully established by genetic interrogation of an axis of autophagy-Nrf2 activation-cardiac remodeling and dysfunction *in vivo*.

In the present study, we verified the critical role of autophagy in controlling Nrf2-mediated dichotomy in PO hearts using CR-Atg5KO and Nrf2KO mice. We demonstrated that autophagy inhibition via CR-Atg5KO wipes out Nrf2-operated defense while activating Nrf2-mediated injuries in PO hearts toward heart failure. We also found that autophagy is required for maintaining ERK-dependent downregulation of Nrf2-driven angiotensinogen expression in cardiomyocytes. These findings provide direct evidence to consolidate the emerging notion that autophagy controls Nrf2-mediated dichotomy in the heart.

## Materials and Methods

### Animals

All animals were kept on a 12-hour light/dark cycle in a temperature-controlled room with ad libitum access to food and water. All animals were treated in compliance with the USA National Institute of Health Guideline for Care and Use of Laboratory Animals. The use of animals and all animal protocols were approved by the Institutional Animal Care and Use Committee (IACUC) at the University of South Carolina, United States. The breeding pairs of Nrf2 heterozygote knockout (Nrf2^+/–^) and floxed Atg5 (Atg5^*fl/fl*^) mice in a C57BL/6J genetic background were purchased from Riken BioResource Research Center, Tsukuba, Japan. The breeding pairs of a transgenic mouse strain harboring a tamoxifen-inducible Cre-fusion protein (MerCreMer) under the control of cardiomyocyte-specific α-myosin heavy-chain promoter (Myh6 or αMHC) in a C57BL/6J genetic background were purchased from JAX. Littermates of wild type (WT) and Nrf2^–/–^ (Nrf2KO) mice were generated by breeding pairs of Nrf2^+/–^ mice as we previously described (Li et al., 2014). Littermates of MerCreMer^+^, Atg5^*fl/fl*^, and MerCreMer^+^::Atg5^*fl/fl*^ mice were produced using the breeding pairs of MerCreMer^+^::Atg5^*fl/*+^ and Agt5^*fl/*+^, which were generated by crossing MerCreMer^+^ with Atg5^*fl/fl*^ mice; and double Nrf2 and CR-Atg5 KO (Nrf2^–/–^::Atg5^–/–^) mice were generated by crossing Nrf2^–/–^ mice with MerCreMer^+^::Atg5^*fl/fl*^ mice as we previously described (Qin et al., 2016).

### Induction and Quantification of Recombination

Tamoxifen (Cat#: T5648, Sigma-Aldrich, St. Louis, MO, United States) was dissolved in warm sunflower seed oil at a concentration of 10 mg/ml and injected intraperitoneally (i.p.) at 20 mg/kg body weight daily (20 mg/kg/d) for 3 weeks to avoid the cardiac Cre toxicity as previously reported (Koitabashi et al., 2009). The induction of recombination with tamoxifen was started in male mice at 6 weeks of age. After a time period of 2 weeks for washing out the tamoxifen, the efficacy of recombination was determined by Western blot analysis of ATG5 expression in WT, MerCreMer^+^, MerCreMer^+^::Atg5^*fl/*+^, and MerCreMer^+^::Atg5^*fl/fl*^ mice which received the tamoxifen induction.

### Transverse Aortic Arch Constriction (TAC)

Male littermates of WT and Nrf2^–/–^ (Nrf2KO) mice at 11 weeks of age were subject to sham or TAC operation for 4 or 8 weeks. Male littermates of WT, MerCreMer^+^, Agt5^*fl/fl*^, and MerCreMer^+^::Atg5^*fl/fl*^ or MerCreMer^+^, MerCreMer^+^::Nrf2KO, MerCreMer^+^Atg5^*fl/fl*^, and MercreMer^+^::Atg5^*fl/fl*^::Nrf2KO mice which received tamoxifen induction as described above were subject to sham or TAC operation at 11–12 weeks of age and euthanized 4 or 6 weeks later. The sham or TAC operation in mice was performed under deep anesthesia as previously described (Li et al., 2009b; Qin et al., 2016). Briefly, mice were anesthetized by i.p. injection of ketamine (80 mg/kg) and xylazine (5 mg/kg). The use of a horizontal incision at the level of the suprasternal notch allows direct visualization of the transverse aorta without entering the pleural space and thus obviates the need for mechanical ventilation. The transverse aorta was banded between the right innominate and left carotid arteries to a 27-gage needle using a 6-0 nylon silk suture. Sham operation on mice were similar but without actual aortic banding and these mice served as a control group for all experimental groups. Cardiac hypertrophy was determined by heart weight–to–tibial length (HW/TIBIA) ratio, heart weight–to–body weight (HW/BW) ratio and expression levels of cardiac hypertrophy marker genes including atrial natriuretic factor (ANF), brain natriuretic peptide (BNP), alpha-myosin heavy chain (α-MHC), beta-myosin heavy chain (β-MHC), sarco-endoplasmic reticulum calcium ATPase2a (SERCA2a).

### Echocardiographic Analysis

Echocardiography was performed on anesthetized mice using the Vevo 2100 High-Resolution Imaging System (VisualSonics Inc.) with a 30-MHz high-frequency linear transducer as previously described (Li et al., 2009b, Li et al., 2014; Qin et al., 2016). Briefly, mice were anesthetized with 3% isoflurane and maintained with 1.5% isoflurane in room air supplemented with 100% O_2_. After the anterior chest was shaved, the animals were placed on a warming pad to maintain normothermia. The echocardiographic gel was warmed before use to avoid hypothermia. Care was taken to avoid excessive pressure on the thorax, which can induce bradycardia. Two-dimensional (2D) long axis images of the left ventricle (LV) were obtained at the plane of the aortic and mitral valves where the LV cavity is largest, and visualization of the LV apex is adequate; and a short-axis image was recorded at the level of the papillary muscles. A 2D guided M-mode echocardiogram was recorded through the anterior and posterior LV walls at 21 frames/s. Images were obtained at the level of the papillary muscle tips, and measurements were then performed to obtain the LV internal dimension (LVID; in mm), interventricular septum thickness (IVS), and LV posterior wall thickness (LVPW; in mm). LV percent fractional shortening FS (%) was calculated via VisualSonics Measurement Software.

### Pathology

Mice were anesthetized and perfused via the LV apex with saline (0.9% NaCl) to wash out the blood from the heart tissue. Then, the hearts were dried on gauze, weighed, dissected, and frozen. Lungs and tibias were also dissected. Lungs were dried on gauze and weighed. The length of the tibia from the condyle to the tip of the medial malleolus was measured by micrometer calipers.

### Histological and Immunochemical Analysis

Hearts were cannulated via the LV apex, cleared of blood by perfusion with normal saline at 90 mmHg, arrested in diastole with 60 mM KCl, fixed by perfusion with 4% paraformaldehyde, and embedded in paraffin. Paraffin sections were prepared (5 μm, Leica RM2235, rotary microtome) and stored at room temperature until staining. For LV cardiomyocyte cross-sectional area, coronal sections were deparaffinized and the cardiomyocyte membranes were stained with Alexa Fluor 488 conjugated wheat germ agglutinin (WGA) (Invitrogen Corp., Carlsbad, CA, United States) and observed using a fluorescence microscope (Nikon Eclipse 80i; Nikon Instruments Inc. Tokyo, Japan) at 400× magnification. Twenty fields of each section were randomly photographed using NIS-Elements F 4.0 imaging software (Nikon Instruments Inc. Tokyo, Japan) and cross-sectional areas of 1,000–1,400 circular cardiomyocytes per heart was measured using Image-Pro Plus software (Media Cybernetics, Inc., Bethesda, MD, United States). For myocardial fibrosis, coronal sections were stained for collagen with a Masson’s Trichrome Kit (Poly Scientific, Bay Shore, NY, United States) according to the protocol provided by the manufacturer. Sections were observed under a light microscope (Nikon Eclipse 80i; Nikon Instruments Inc. Tokyo, Japan) at 200× magnification. Twenty fields of each section were randomly photographed using NIS-Elements F 4.0 imaging software (Nikon Instruments Inc. Tokyo, Japan). The percentage of fibrosis (the blue stained area) was measured by Image-Pro Plus software (Media Cybernetics, Inc., Bethesda, MD, United States). At least two sections of each heart were analyzed for the measurements of cardiomyocyte cross sectional areas and cardiac fibrosis. Sub-cellular locations of p-ERK and Nrf2 were analyzed by immunochemical staining using anti-p-ERK (cat#: 9101, Cell Signaling Technology, Inc., Danvers, MA, United States) and anti-Nrf2 (cat#: sc-722, 1:200, Santa Cruz Biotechnology Inc., Dallas, TX, United States). Two sections from each heart were analyzed.

### Cell Cultures, Adenoviral Infection, Oligo and Plasmid Transfection

Rat neonatal cardiac myocytes were isolated and cultured as previously described (Li et al., 2009b; Qin et al., 2016). Adenovirus of control scramble shRNA (Ad-shCtr) and rat Nrf2 shRNA (Ad-shNrf2) were generated as previously reported (Li et al., 2009b). Rat neonatal cardiomyocytes were infected with Ad-shCtr (20 MOI) and Ad-shNrf2 (20 MOI) in serum-free DMEM for 6 h and the cultured with full growth medium (1 g/L glucose DMEM supplemented with 8% horse serum (HS) and 5% newborn calf serum (NCS) for additional 24 h. The infected cardiomyocytes were further transfected with scramble siRNA against luciferase (siCtr, 5′-CGUACGCGGAAUACUUCGATT-3′, purchased from Invitrogen Corp., Carlsbad, CA, United States), Agt5 siRNA (siAtg5-1: 5′-GACGCUGGUAACUGACAAATT-3′, siAtg5-2: 5′-GUCAGGUGAUCAACGAAAUTT-3′, or siAtg5-3: 5′-CCACAACUGAACGGCCUUUTT-3′, purchased from Ribobio, Guangzhou, China) using Lipofectamine 2000 (Cat#: 12566-014, Thermo Fisher Scientific, Waltham, United States) for 6 h and then cultured with full growth medium aforementioned for 24 h. The transfection was repeated once and then the cells were serum starved for 48 h prior to the treatment with Ang II (1 μM, Cat#: RAB0010, Sigma-Aldrich, St. Louis, MO, United States) and U0126 (1 μM, an ERK inhibitor, Cat#: U120, Sigma-Aldrich, St. Louis, MO, United States) in serum free DMEM. Based on our pilot study that Ang II stimulation induced phosphorylation of ERK at 10 min and upregulation of LC3-II at 24 h while enhancing protein expression of angiotensinogen at 48 h in rat neonatal cardiomyocytes (data not shown), we treated the cardiomyocytes with Ang II for 10 min prior to Western blot analysis of ERK phosphorylation and 24 h prior to Western blot analysis of LC3-II and p62 protein expression, and 48 h prior to Western blot analysis of angiotensinogen protein expression. These experiments were repeated for four times.

### Reverse Transcription-Polymerase Chain Reaction (RT-PCR) and Quantitative Real Time (qPCR)

The total RNA from the LV was extracted using RNeasy Fibrous Tissue Mini kit (Qiagen Inc., Valencia, CA, United States), and the reverse transcription reaction was performed with 1 μg of total RNA using a RevertAid^TM^ First Strand cDNA Synthesis Kit (Cat#: K1622, Thermo Scientific). qPCR was carried out using the Bio-Red CFX96^TM^ Real-Time system (C1000^TM^ Thermal Cycler, Bio-Red Laboratories, Inc. Hercules, CA, United States). Expression levels of target genes were normalized by concurrent measurement of glyceraldehyde-3-phosphate dehydrogenase (GAPDH) mRNA levels. Genomic DNAs were also extracted from mouse tails and subjected to PCR for genotyping of transgene mice. Primers that were used for PCR are summarized in Supplementary Table S1.

### Western Blot Analysis

Left ventricle or cardiomyocyte whole lysates or nuclear and cytoplasmic fractions of cardiomyocytes extracted with a NE-PER Nuclear and Cytoplasmic Extraction Kit (Cat#: 78833, ThermoFisher Scientific, United States) were subject to Western blot analysis as we previously described.(Li et al., 2009b, Li et al., 2014; Qin et al., 2016) Antibodies used included anti-APG5L/ATG5 monoclonal antibody (Cat#: 3447-1, Abcam, Cambridge, United Kingdom), anti-LC3B polyclonal antibody (Cat#: L7543, Sigma-Aldrich, St. Louis, MO, United States), anti-ERK antibody (Cat#: 9101S, Cell Signaling Technology, United States), anti-angiotensinogen (Cat#: sc-7419, Santa Cruz Biotechnology Inc., Dallas, TX, United States), anti-p62 (Cat#: ab91526, Abcam, Cambridge, United Kingdom), anti-NQO1 monoclonal antibody (Cat#: sc-376023, Santa Cruz Biotechnology, Inc., Dallas, Texas, United States), anti-Nrf2 polyclonal antibody (Cat#: sc-722, Santa Cruz Biotechnology, Inc., Dallas, TX, United States), anti-GAPDH polyclonal antibody (Cat#: G9545, Sigma-Aldrich, St. Louis, MO, United States), peroxidase-conjugated AffiniPure goat anti-Mouse IgG (H+L) (Cat#: ZB2305, ZSGB-BIO, Beijing, China), and peroxidase-conjugated AffiniPure rabbit anti-goat IgG (H+L) (Cat#: ZB2306, ZSGB-BIO, Beijing, China).

### Statistics

Data are shown as mean ± SD. Differences between 2 groups were evaluated for statistical significance using the Student t test. When differences among >3 groups were evaluated, results were compared by one-way ANOVA with Bonferroni test for multiple comparisons. Survival rate between experimental groups after TAC was analyzed using Kaplan Meier test. Differences were considered significant at *P* < 0.05.

## Results

### Cardiac Autophagy Protects Against PO-Induced Cardiomyopathy and Controls Nrf2-Mediated Dichotomy in PO Hearts

To determine a causative role of autophagy in the regulation of cardiomyopathy and Nrf2-mediated dichotomy in PO hearts, we determined the impact of cardiac specific autophagy inhibition via CR-Atg5KO on TAC-induced cardiac remodeling and dysfunction as well as Nrf2-mediated dichotomy (Qin et al., 2016) in adult mice. In our pilot study, we found that the expression of Cre *per se* induced by the nontoxic doses of tamoxifen established by a previous study (Koitabashi et al., 2009) did not have negative impact on the heart or cause any other health issues at the basal conditions, but it did slightly worsen TAC-induced cardiomyopathy (data not shown). To minimize the impact of off-target effects of MerCreMer, we used mice which all carried the MerCreMer transgene and all received the same regime of tamoxifen treatment.

**FIGURE 1:**
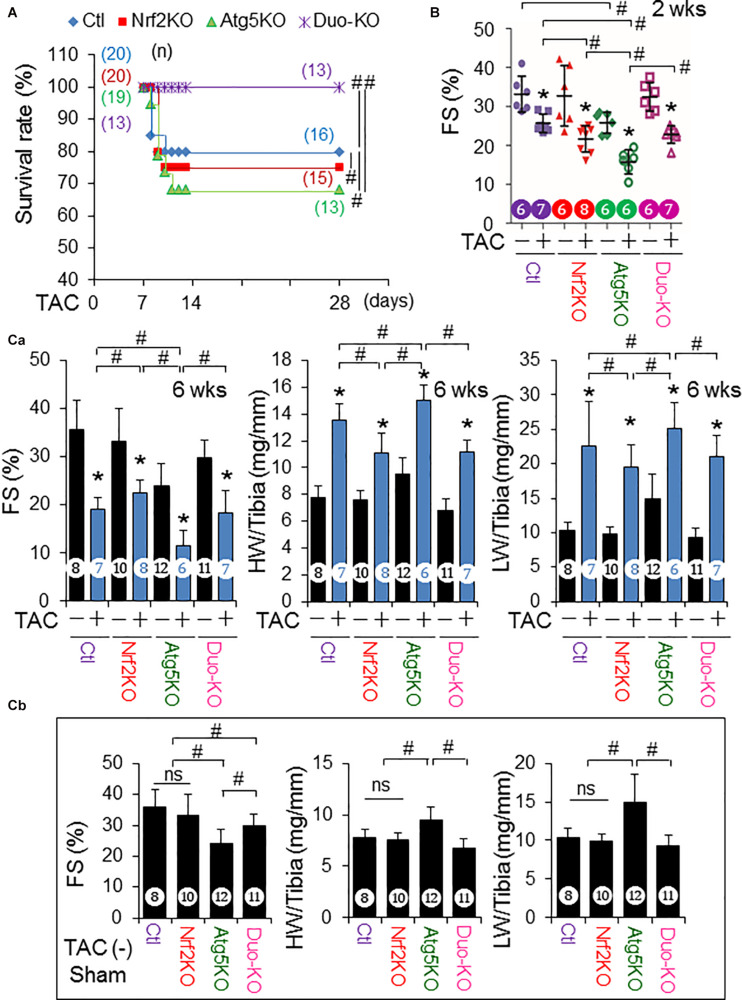
Nrf2-mediated dichotomy in mice in response to pressure overload. The mice with genotypes of MerCreMer^+^ (Ctl), MerCreMer^+^::Nrf2 KO (Nrf2KO), MerCreMer^+^::Atg5^*fl/fl*^ (Atg5KO), and MerCreMer^+^::Atg5^*fl/fl*^::Nrf2KO (Duo-KO) received tamoxifen treatment as described in the section “Materials and Methods” and then were subject to sham and TAC operation for 6 weeks. **(A)** Survival rate at 4 weeks after TAC. ^#^*p* < 0.05 by Kaplan Meier test. **(B)** FS (%) at 2 weeks after TAC. *p < 0.05 vs TAC (–) in the same groups using the Student *t* test; ^#^*p* < 0.05 between indicated groups using one-way ANOVA followed by Bonferroni test. **(C)** FS (%), HW/Tibia ratio and LW/Tibia ratio at 6 weeks after TAC. *p < 0.05 vs TAC (–) in the same groups using the Student *t* test; ^#^*p* < 0.05 between indicated groups using one-way ANOVA followed by Bonferroni test. (a) Statistic analysis of both sham and TAC groups; (b) Statistic analysis of sham groups alone. Animal number for each group is indicated in each figure. The efficacy of Cre and LoxP recombination which ablates myocardial Atg5 expression in Atg5KO and Duo-KO mice was confirmed by Western blot analysis.

**FIGURE 2:**
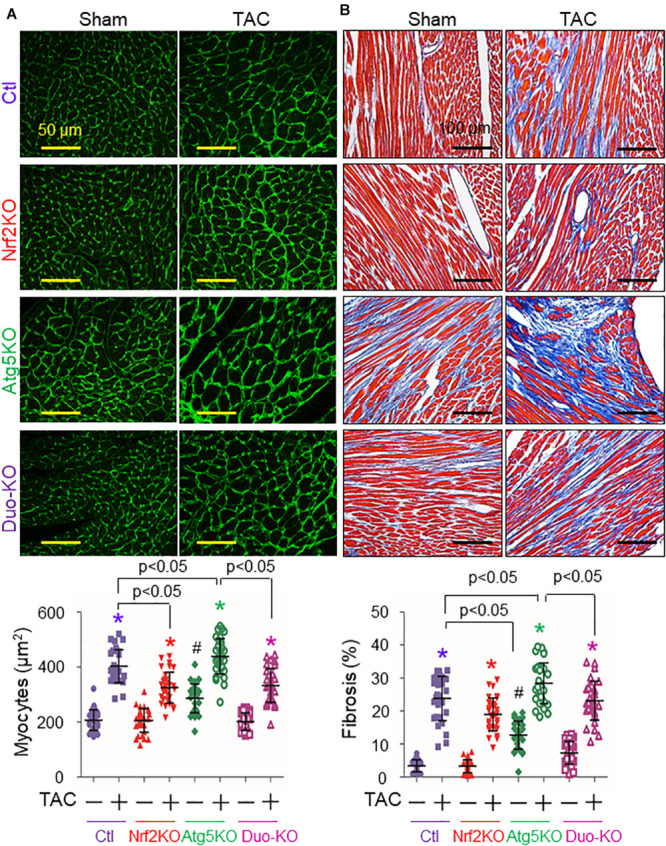
Nrf2-mediated cardiac pathological remodeling in mice in response to pressure overload. The mice with indicated genotypes received tamoxifen treatment as described in the section “Materials and Methods” and then were subject to sham and TAC operation for 6 weeks. **(A)** Cardiomyocyte sizes. **(B)** Cardiac fibrosis. Animal number for each group is 3∼4. *p < 0.05 vs the sham, TAC (–) in the same groups using the Student *t* test; ^#^*p* < 0.05 vs TAC (–) in all groups using the Student *t* test; *p* < 0.05 between indicated groups using one-way ANOVA followed by Bonferroni test.

Transverse aortic arch constriction caused around 20% mortality in Control MerCreMer^+^ (Ctl) mice as described elsewhere (Qin et al., 2016) and it was slightly increased by additional Nrf2KO (25%) (Figure 1A), suggesting Nrf2-mediated protection. Compared with the Ctl or Nrf2KO groups, CR-Atg5KO (32%) enhanced TAC-induced death; however, the CR-Atg5KO-enhanced death was reversed by additional Nrf2KO (Figure 1A), demonstrating autophagy inhibition-dependent activation of Nrf2-mediated detrimental effects. In addition, when cardiac autophagy flux is intact within the first 2 weeks after TAC (Qin et al., 2016), Nrf2KO enhanced TAC-induced cardiac dysfunction (Figure 1B and Supplementary Table S2) as we previously reported (Li et al., 2009b). When cardiac autophagy is inhibited at 6 weeks after TAC (Qin et al., 2016), however, Nrf2KO improved TAC-induced cardiac dysfunction (Figure 1Ca and Supplementary Table S3) and attenuated TAC-induced cardiac hypertrophy and fibrosis (Figure 2 and Supplementary Table S3) as we previously reported (Qin et al., 2016). On the other hand, CR-Atg5KO resulted in declined cardiac function in sham-operated mice and exaggerated TAC-induced cardiac dysfunction at both 2 and 6 weeks (Figures 1B,Ca,b and Supplementary Tables S2, S3). Also, CR-Atg5KO exacerbated TAC-induced cardiac hypertrophy and fibrosis at 6 weeks (Figure 2 and Supplementary Table S3). These results demonstrate an essential role of cardiac autophagy in maintaining cardiac homeostasis at both basal and PO conditions. Notably, all these adverse impacts of CR-Atg5KO could be rescued by additional Nrf2KO (Figures 1, 2 and Supplementary Tables S2, S3), demonstrating that autophagy inhibition activates Nrf2-mediated damage in stressed hearts, such as PO hearts. To this end, we demonstrate that Cre (MerCreMer) expression *per se* has less impact on Nrf2-mediated dichotomy in PO hearts. Accordingly, we validate our previous findings (Li et al., 2009b; Qin et al., 2016) but give in addition, direct evidence to consolidate the concept that autophagy controls Nrf2-mediated dichotomy in PO hearts.

### Autophagy Inhibition-Induced Inactivation of ERK Is an Upstream Signal to Enhance Nrf2-Operated Upregulation of Angiotensinogen in PO Hearts

We have demonstrated that autophagy inhibition enables Nrf2 to upregulate myocardial expression of angiotensinogen, a primary cause of cardiac pathological remodeling and dysfunction (Wollert and Drexler, 1999), in PO hearts (Qin et al., 2016). At the molecular level, autophagy impairment suppresses activation of Jak2/Fyn pathway which operates Nrf2 nuclear export for degradation in PO hearts (Qin et al., 2016). A previous study showed that the autophagosomal membrane could serve as a platform to regulate intracellular signaling transduction such as the activation of extracellular signal regulated kinase (ERK) (Martinez-Lopez et al., 2013). Given that autophagy are intertwined with multiple kinase pathways (Kroemer et al., 2010) including mitogen activated protein kinases (MAPKs), such as ERK, and protein kinase B (AKT) cascades, all of which regulate Nrf2 activity (Li et al., 2009a; Bryan et al., 2013), we questioned whether these kinases are also involved in the emerging axis of autophagy inhibition-Nrf2-angiotensinogen expression in PO hearts.

**FIGURE 3:**
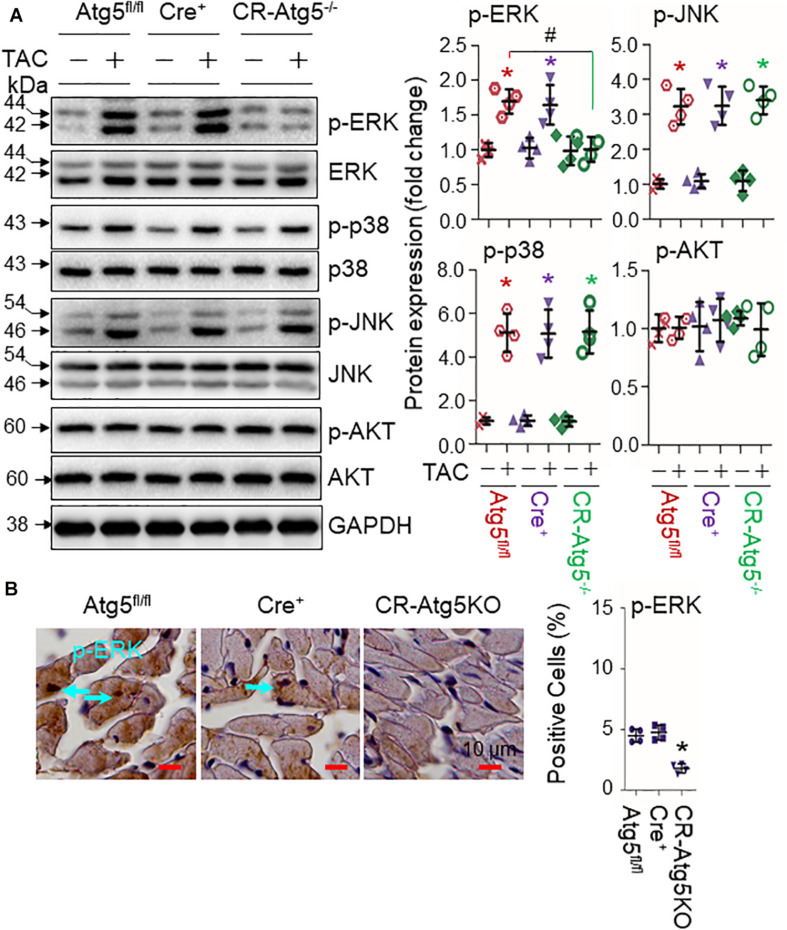
The impact of cardiomyocyte-restricted knockout of Atg5 on pressure overload-induced activation of MAPK and expression of angiotensinogen (Agt) in the heart. Male floxed Atg5 (Atg5^*fl/fl*^), MerCreMer^+^ (Cre^+^), and MerCreMer^+^::Atg5^*fl/fl*^ (CR-Atg5^–/–^ or CR-Atg5KO) after tamoxifen-induced ablation of Atg5 were subject to sham or TAC operations for 4 weeks. **(A)** Western blot analysis of LV tissues (*n* = 4). The right panel is the semi-quantified densitometry analyses. *p < 0.05 vs TAC (–) in the same groups using the Student *t* test; ^#^*p* < 0.05 between the indicated groups using one-way ANOVA followed by Bonferroni test. **(B)** Immunohistochemical staining of phosphorylated ERK and Nrf2 in pressure overloaded hearts of Atg5^*fl/fl*^, MerCreMer^+^ (Cre^+^) and CR-Atg5KO mice. LV tissue sections of mice with indicated genotypes were stained with antibodies for phosphorylated ERK (p-ERK) and Nrf2 (data not shown).

**FIGURE 4:**
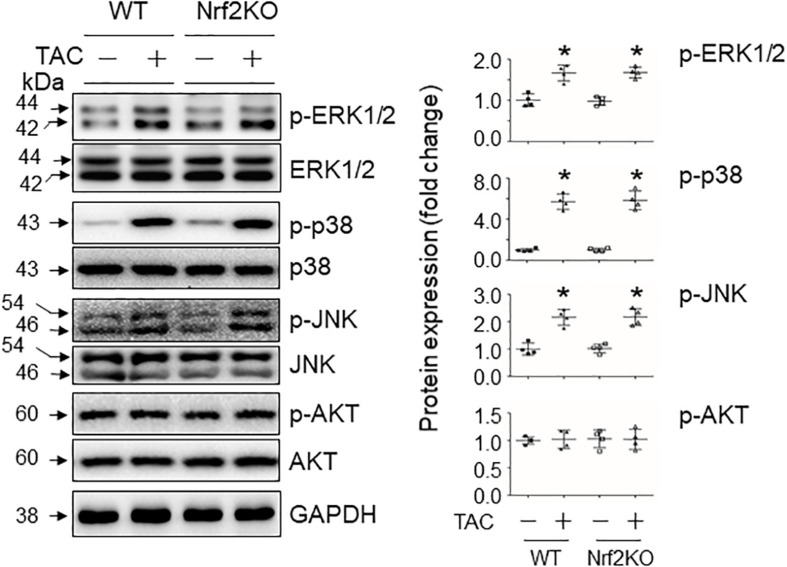
The impact of Nrf2 knockout on pressure overload-induced activation of MAPKs and AKT in the heart. Male WT and Nrf2 knockout (Nrf2KO) mice at age of 9∼10 weeks were subject to sham or TAC operations for 4 weeks and then left ventricles of these mice were harvested for Western blot analyses of MAPKs and AKT activities (*n* = 4). *p < 0.05 vs TAC (–) in the same groups using the Student *t* test; *p* < 0.01 between the indicated groups using one-way ANOVA followed by Bonferroni test.

In 4-week TAC hearts in which the axis of Nrf2-angiotensinogen activation is boosted by CR-Atg5KO (Qin et al., 2016), we found that such autophagy inhibition minimally regulated the activities of MAPK p38 and JNK or AKT, but strongly suppressed the activities of ERK (Figure 3A). On the other hand, Nrf2KO did not affect PO-induced activation of these kinases (Figure 4) but downregulated PO-induced upregulation of angiotensinogen expression at 4 weeks (data not shown) when cardiac autophagy flux declines as we previously observed (Qin et al., 2016). These results indicate that proper initiation of autophagy, e.g., autophagosome formation, is critical for signal-induced activation of ERK in cardiomyocytes as observed in the other cell types (Martinez-Lopez et al., 2013). The autophagy-operating ERK activity is likely an upstream event of Nrf2-mediated regulation of angiotensinogen expression in the heart.

To further establish a pathophysiological relevance of the autophagy inhibition-ERK-Nrf2 axis, we studied the nuclear location of ERK and Nrf2 in PO hearts. Immunochemical staining showed that there is no detectable level of nuclear phosphorylated ERK (p-ERK) or Nrf2 in 4-week sham hearts of Atg5^*fl/fl*^, MerCreMer^+^ and CR-Atg5KO mice (data not shown). However, 4-week TAC led to increased levels of nuclear p-ERK in the hearts of control Atg5^*fl/fl*^ and MerCreMer^+^ mice but not in the hearts of CR-Atg5KO mice (Figure 3B). Also, 4-week TAC led to increased levels of nuclear Nrf2 in the hearts of control Agt5^*fl/fl*^ and MerCreMer^+^ mice and it was more dramatic in the hearts of CR-Atg5KO mice (data not shown) as we previously observed (Qin et al., 2016). Taken together, this reciprocal relationship between nuclear p-ERK and Nrf2 in PO hearts, as well as the enhanced nuclear Nrf2 levels in autophagy deficient cardiomyocytes suggest that not only inactivation of Jak/Fyn pathway (Qin et al., 2016) but also ERK is responsible for the nuclear accumulation of Nrf2 leading to upregulation of angiotensinogen in autophagy insufficient myocardium.

### Autophagy Inhibition Suppresses ERK-Mediated Nrf2-Driven Angiotensinogen Expression in Cardiomyocytes

**FIGURE 5:**
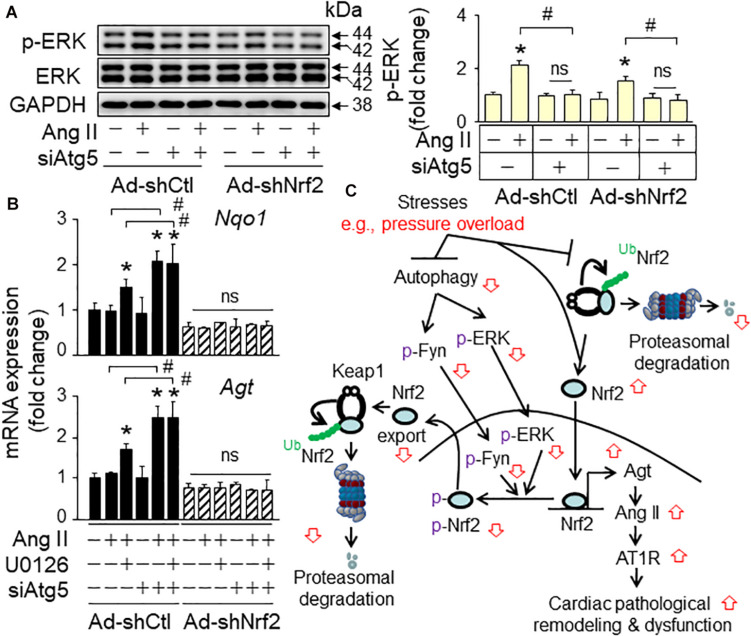
The impact of autophagy inhibition on Ang II-induced ERK activation, Nrf2 activity and angiotensinogen (Agt) expression in the primary culture of neonatal rat ventricular myocytes (NRVMs). NRVMs were infected with adenovirus of scramble (Ad-shCtl) or Nrf2 shRNA (Ad-shNrf2) and then transfected with the control siLuc (siCtl) or Atg5 siRNA (siAtg5) prior to the treatment of Ang II (1 μM) and U0126 (1 μM) as described in the section “Materials and Methods.” **(A)** Representative immunoblots and densitometric analysis (*n* = 4). *p < 0.05 vs the vehicle control (–) treated Ad-shCtl + siCtl cells using the Student *t* test; ^#^*p* < 0.05 between indicated groups using one-way ANOVA followed by Bonferroni test. ns, non-significant. **(B)** qPCR analysis of Nqo1 and angiotensinogen (Agt) expression (*n* = 4). *p < 0.05 vs the vehicle control (–) group using the Student *t* test; ^#^*p* < 0.05 between indicated groups using one-way ANOVA followed by Bonferroni test. **(C)** A working hypothesis. Sustained pressure overload interrupts Keap1-mediated Nrf2 ubiquitination for proteasomal degradation, thus increasing free Nrf2 which is translocated into nuclei and activates its target gene expression. On the other hand, it also induces autophagy inhibition to switch off Jak/Fyn and ERK-mediated phosphorylation of nuclear Nrf2 for nuclear export and degradation, which in turn results in enhancement of Nrf2-driven expression of Agt, leading to pathological activation of Ang II-AT1R axis in the heart. Accordingly, sustained pressure overload causes autophagy impairment, which in turn intensifies Nrf2-mediated cardiac pathological remodeling and dysfunction toward heart failure. Ub, ubiquitin; up arrow, increased activity; down arrow, decreased activity.

To establish the signaling axis of autophagy inhibition-ERK inactivation-Nrf2 activation-angiotensinogen expression in PO hearts, we determined the effect of angiotensin II (Ang II) on ERK activation, Nrf2 activity and angiotensinogen expression in primary culture of neonatal rat ventricular myocytes (NRVMs) with a combination of Atg5 and/or Nrf2 knockdown and/or ERK inhibition. As shown in Figure 5A, Ang II induced phosphorylation of ERK along with increased LC3 and angiotensinogen expression (data not shown) as we previously observed (Qin et al., 2016) in the control group. These results indicate that Ang II-induced upregulation of angiotensinogen associates with the activation of autophagy and ERK in cardiomyocytes. Knockdown of ATG5 alone resulted in downregulation of LC3-II and upregulation of p62 (data not show) as we previously observed (Qin et al., 2016), indicating impaired autophagy in cardiomyocytes. The ATG5 knockdown-induced autophagy inhibition blocked the Ang II-induced activation of ERK whereas dramatically enhancing the Ang II-induced angiotensinogen expression (Figures 5A,B), demonstrating that autophagy inhibition suppresses activation of ERK while facilitating upregulation of angiotensinogen in cardiomyocytes as observed in autophagy-impaired PO hearts (Qin et al., 2016). Knockdown of Nrf2 alone had minimal impact on the Ang II-induced activation of ERK but suppressed the Ang II-induced upregulation of angiotensinogen (Figures 5A,B). Although knockdown of Nrf2 did not affect the ATG5 deficiency-induced inactivation of ERK, it blocked the ATG5 deficiency-induced enhancement of angiotensinogen expression (Figures 5A,B). These results demonstrate that Nrf2-mediated angiotensinogen expression in cardiomyocytes (Qin et al., 2016) is negatively regulated in part by autophagy-dependent ERK activation, supporting that ERK signaling plays an important role in the control of Nrf2-mediated upregulation of angiotensinogen in autophagy-impaired hearts.

To establish a causative link between ERK, Nrf2 activation, and angiotensinogen expression in cardiomyocytes, we used an ERK inhibitor, U0126 in the primary culture of NRVMs. U0126 enhanced Ang II-induced upregulation of NAD(P)H quinone dehydrogenase 1 (Nqo1), a typical Nrf2 target gene and angiotensinogen mRNA levels in control group (Figure 5B). These results reveal that ERK serves a negative regulator of Nrf2 activation and angiotensinogen expression when autophagy is intact. Notably, knockdown of ATG5 dramatically enhanced Ang II-induced expression of Nqo1 and angiotensinogen mRNAs and this enhancement could not be further increased by U0126; whereas, both U0126-potentiated and ATG5 deficiency-augmented angiotensinogen expression were blocked by knockdown of Nrf2 (Figure 5B). These results indicate that ERK suppresses angiotensinogen expression by inactivating Nrf2, serving as a negative feedback mechanism for the control of angiotensinogen expression in autophagy intact cardiomyocytes; however, autophagy inhibition turns off the negative feedback regulation thus exaggerating angiotensinogen expression in cardiomyocytes.

## Discussion

In the present study, we provided a few pieces of evidence which help clarify the contradictory roles of autophagy and Nrf2 in cardiac remodeling and dysfunction, thereby consolidating the notions that (1) autophagy activation is cardioprotective; (2) autophagy is essential for Nrf2-mediated cardiac protection; (3) autophagy inhibition or impairment switches on Nrf2-operated myocardial damage; and (4) autophagy inhibition activates Nrf2-driven transcription of angiotensinogen in cardiomyocytes, thereby contributing to the pathological activation of Ang II-Ang II receptor type 1 signaling axis in the heart (Wollert and Drexler, 1999) (Figure 5C).

### Does Autophagy Activation Protect Against PO-Induced Cardiomyopathy?

Although not entirely conclusive, most of the recent studies have revealed that autophagy activation is most likely an adaptive response in PO hearts (Wang and Cui, 2017; Sciarretta et al., 2018). Of note, we lately provided direct evidence to support this notion, i.e., that enhancement of myocardial autophagy via CR-Atg7 transgenic overexpression dramatically ameliorated PO-induced cardiomyopathy and heart failure (Qi et al., 2020). However, the adaptive nature of autophagy activation in PO hearts may not be completely addressed without a CR autophagy inhibition approach. This is likely due to experimental weaknesses of the original study which demonstrated a cardioprotective role of autophagy using MerCreMer^+^::Atg5^*fl/fl*^ mice after tamoxifen induction (CR-Atg5KO) in adult (Nakai et al., 2007). In this pioneer study, it is highly possible that the observed cardiac hypertrophy and dysfunction of CR-Atg5KO mice at the basal line are caused by Cre-mediated cardiotoxicity (Koitabashi et al., 2009) due to the high dose injection of tamoxifen (80 μg per g body weight, daily, for one week) (Nakai et al., 2007). In addition, Atg5^*fl/fl*^, but not MerCreMer^+^ mice were used as the control (Nakai et al., 2007), which may cover the Cre-mediated myocardial damage while amplifying the CR-Atg5KO-dependent adverse phenotypes. In the present study, however, we addressed all of these concerns by using mice that all carried MerCreMer transgene and all received the same regime of tamoxifen treatment which has minimal impact on the heart at least at basal line condition (Koitabashi et al., 2009) to minimize the known and yet unknown off-target effects of MerCreMer on the heart. Importantly, compared with such rigorous control of MerCreMer^+^ mice, we found that CR-Atg5KO mice developed more severe cardiac hypertrophy and dysfunction at both 2 and 6 weeks after TAC, demonstrating a cardioprotective role of autophagy in PO hearts. Even in sham operated mice, we observed that CR-Atg5KO led to cardiac hypertrophy and dysfunction, indicating a housekeeping role of autophagy in suppressing cardiac maladaptive remodeling and dysfunction. Taken together, our results consolidate the notions that autophagy plays a critical role in maintaining cardiac homeostasis and autophagy activation is an adaptive response in PO hearts.

### Does Autophagy Controls Nrf2-Mediated Dichotomy in PO Hearts?

Nrf2 has been considered as a hermetic factor (Maher and Yamamoto, 2010). However, such a theory that either too much or too little of Nrf2 can lead to pathological endpoints cannot explain Nrf2-mediated cardiac phenotypes (Zang et al., 2020a). For example, CR-Nrf2 Tg mice is perfectly normal under physiological conditions, and sustained PO does not result in super-activation of Nrf2 in the heart although Nrf2 activation promotes the PO-induced cardiomyopathy (Zang et al., 2020a). In the present study, we showed that cardiac specific autophagy inhibition via CR-Atg5KO wiped out Nrf2-mediated cardiac protection while turning on Nrf2-mediated pathological cardiac remodeling and dysfunction, providing direct evidence to demonstrate a critical role autophagy in the control of Nrf2-mediated dichotomy in PO hearts. These findings also indicate that Nrf2 does not act as a hermetic factor and Nrf2-mediated dichotomous effects are coupled with specific cellular processes, such as autophagy, in the heart.

### How Does Autophagy Control Nrf2-Mediated Dichotomy in PO Hearts?

Our previous study have shown that autophagy inhibition inactivates Jak/Fyn signaling for Nrf2 nuclear export and degradation, leading to nuclear accumulation of Nrf2 to drive angiotensinogen expression in cardiomyocytes, which inevitably activates pathological Ang II-Ang II receptor type 1 (AT1R) signaling axis in the heart (Wollert and Drexler, 1999). In this study, we further demonstrated that autophagy inhibition inactivated ERK, thereby activating the Nrf2-angiotensinogen axis in cardiomyocytes. These results not only validate our prior findings, but also give more mechanistic insights into autophagy-dependent control of Nrf2 signaling in PO hearts. However, how exactly autophagy controls Jak/Fyn and/or ERK signaling for the negative control of Nrf2-operated angiotensinogen expression in PO hearts has not been completely delineated. The critical downstream effectors of Nrf2-mediated myocardial damage and dysfunction in autophagy-impaired PO hearts remain to be determined. Recently, we demonstrated that CR-Atg5KO exacerbates Nrf2-mediated cardiac pathological remodeling and dysfunction and intensifies Nrf2-driven transcription of a subset of genes, such as acyl-CoA synthetase long-chain family 4 (Acsl4), cluster of differentiation 36 (Cd36), angiotensinogen (Agt), and Kruppel-like factor 9 (Klf9), while suppressing Nrf2-operated transcription of cellular defense genes including glutathione peroxidase 4 (Gpx4) and ferroptosis suppressor protein 1 (Fsp1), also known as apoptosis-inducing factor mitochondria 2 (Aifm2), thereby exaggerating the progression of cardiomyopathy associated with type 1 diabetes (Zang et al., 2020b). Whether such autophagy-governed coordination of Nrf2 signaling pathways is also disturbed in PO hearts deserves investigations, thus providing mechanistic insights into the molecular network by which autophagy controls Nrf2-mediated dichotomy in the heart.

### What Are Clinical Relevance and Limitations of This Study?

The enthusiasm for activating Nrf2 as a novel approach to treat human diseases, at least non-cardiac disease, remains very high; a number of clinical trials of various phases on Nrf2 activators for treating diseases such as diabetic complications and cancers are still actively ongoing (Al-Sawaf et al., 2015). This study clearly demonstrated autophagy inhibition-dependent activation of Nrf2-mediated cardiac damage and dysfunction. Accordingly, it raises a concern regarding the ‘dark’ side of Nrf2 in these clinical therapies, especially when the treated subjects are compounded with conditions such as hypertensive, ischemic and diabetic cardiomyopathies, all of which likely have myocardial autophagy inhibition (Wang and Cui, 2017; Zang et al., 2020a). The therapeutic efficacy of targeting Nrf2 for these diseases may not be achieved without shutting down Nrf2-mediated adverse signaling in the heart. These issues further create the need for better understanding of the molecular mechanisms underlying such unusual coupling between autophagy inhibition and detrimental activation of Nrf2 in stressed hearts, which have not been completely dissected in the present study. Whether such coupling contributes to PO-induced cardiac diastolic dysfunction has also remained to be addressed. These limitations need further investigation, thereby uncovering the nature of the unique coupling between autophagy function and Nrf2 signaling in cardiac pathological remodeling and dysfunction. As a result, the outcome may provide new insights into the development of a novel therapeutic approach which could simultaneously activate autophagy and Nrf2 for treatment of chronic heart diseases with cardiac autophagy inhibition.

## Data Availability Statement

The raw data supporting the conclusions of this article will be made available by the authors, without undue reservation.

## Ethics Statement

The animal study was reviewed and approved by Kenneth Walsh, IACUC of University of South Carolina.

## Author Contributions

XW and TC conception, design, and grant support of this research. MN and PN grant support of this research. WWu, QQ, YD, HZ, D-SL, and WWa performed experiments and analyzed data. WWu, QQ, YD, and TC interpreted results of experiments and prepared figures. WWu, XW, MN, PN, and TC wrote the manuscript. All authors approved the final version of the manuscript.

## Conflict of Interest

The authors declare that the research was conducted in the absence of any commercial or financial relationships that could be construed as a potential conflict of interest.
