# Supplementary material for: Autophagy Controls Nrf2-Mediated Dichotomy in Pressure Overloaded Hearts
Source: Front Physiol. 2021 May 13;12:673145. doi: 10.3389/fphys.2021.673145 (PMC8155729; doi:10.3389/fphys.2021.673145)
Supplement: Supplementary file 1 [file Data_Sheet_1.pdf]

Autophagy controls Nrf2-mediated dichotomy in pressure overloaded hearts

Weiwei Wu[1], Qingyun Qin[2], Yan Ding[3], Huimei Zang[1], Dong-Sheng Li[3], Mitzi Nagarkatti[4], Prakash Nagarkatti[4], Wenjuan Wang[5], Xuejun Wang[6*], Taixing Cui[1*]

[1]Department of Cell Biology and Anatomy, [4]Department of Pathology, Microbiology and Immunology, University of South Carolina School of Medicine, Columbia, SC 29208, USA

[2]Department of Cardiology, Tianjing First Central Hospital, Tianjing 300192, P.R. China

[3]Hubei Key Laboratory of Embryonic Stem Cell Research, Taihe Hospital, Hubei University of Medicine, Shiyan, Hubei, 442000, China

[5]Vascular Biology Center and Department of Pharmacology and Toxicology, Medical College of Georgia, Augusta University, GA 30912, USA

[6]Division of Basic Biomedical sciences, University of South Dakota Sanford School of Medicine, Vermillion, SD 57069, USA

Corresponding Author:

Dr. Xuejun Wang, Division of Basic Biomedical sciences, University of South Dakota Sanford School of Medicine, Vermillion, SD 57069, USA, Email: xuejun.wang@usd.edu

Dr. Taixing Cui, Department of Cell Biology and Anatomy, University of South Carolina School of Medicine, Columbia, SC 29208, USA., USC-SOM, CBA, Bldg1, Rm36, 6439 Garners Ferry Road, Columbia, SC 29209, USA., Email: taixing.cui@uscmed.sc.edu

Supplementary materials

Table S1. Primers for qPCR and genotyping

Primers	Gene access #	Forward (5'—3')	Reverse (5'—3')	Product
qPCR				
ANF	NM_008725.2	CATCACCCTGGGCTTCTTCCT	TGGGCTCCAATCCTGTCAATC	405
BNP	NM_008726.4	GCGGCATGGATCTCCTGAAGG	CCCAGGCAGAGTCAGAAACTG	418
α-MHC	NM_010856.3	CCAATGAGTACCGCGTGAA	ACAGTCATGCCGGGATGAT	254
β-MHC	NM_080728.2	ATGTGCCGGACCTTGGAA	CCTCGGGTTAGCTGAGAGATCA	170
SERCA2a	NM_009722.3	CCATCTGCTTGTCCATGTCACT	CAAATGGTTTAGGAAGCGGTTACT	213
Nrf2	NM_010902.3	ATGATGGACTTGGAGTTGCC	TCCTGTTCCTTCTGGAGTTG	200
NQO-1	NM_008706.5	CGGTATTACGATCCTCCCTCAACA	AGCCTCTACAGCAGCCTCCTTCAT	120
Agt	NM_007428.3	TTGTCTAGGTTGGCGCTGAA	GGGTGGATGTATACGCGGTC	143
GAPDH	XM_001479322	ATGTTCCAGTATGACTCCACTCACG	GAAGACACCAGTAGACTCCACGACA	171
Genotyping				
Myh6-MerCreMer+	Cre	AGGTGGACCTGATCATGGAG	ATACCGGAGATCATGCAAGC	440
	NW_001030719.1 (Int CTL)	GTAGGTGGAAATTCTAGCATCATCC	CTAGGCCACAGAATTGAAAGATCT	324
floxedAtg5 ($Atg5^{fl/fl}$)	flox	ACAACGTCGAGCACAGCTGCGCAAGG	GTACTGCATAATGGTTTAACTCTTGC	700
	NT_039492.8 (WT)	GAATATGAAGGCACACCCCTGAAATG	GTACTGCATAATGGTTTAACTCTTGC	350
$Nrf2^{-/-}$	LacZ(KO)	TGGACGGGACTATTGAAGGCTG	GCGGATTGACCGTAATGGGATAGG	400
	NM-010902.3 (WT)	TGGACGGGACTATTGAAGGCTG	GCCGCCTTTTCAGTAGATGGAGG	734

Int CTL: internal control (interleukin-2); ANF, atrial natriuretic factor; BNP, B-type natriuretic peptide; α-MHC, alpha-myosin heavy chain; β-MHC, beta-myosin heavy chain; SERCA2a, sarcoplasmic reticulum calcium ATPase2a; NQO-1, NAD(P)H dehydrogenase, quinone-1; Agt, angiotensinogen.

Table S2. Echocardiography of MerCreMer^{+} (Ctl), MerCreMer^{+}::Nrf2KO (Nrf2KO), MerCreMer^{+}::Atg5$^{fl/fl}$ (Atg5KO), and MerCreMer^{+}Atg5$^{fl/fl}$::Nrf2KO (Duo-KO) mice at 2 weeks after TAC.

	**Ctl**		**Nrf2KO**		**Atg5KO**		**Duo-KO**	
	Sham	TAC	Sham	TAC	Sham	TAC	Sham	TAC
(n)	(6)	(7)	(6)	(8)	(6)	(6)	(6)	(7)
IVS;d (mm)	0.83±0.11	1.12±0.10^{A}	0.82±0.12	1.19±0.11^{A}	0.84±0.11	1.17±0.14^{A}	0.84±0.05	1.17±0.11^{A}
LVID;d (mm)	3.56±0.23	4.04±0.18^{A}	3.60±0.29	4.24±0.25^{A}	4.02±0.23	4.49±0.17^{A,B}	3.70±0.27	4.06±0.19^{A,C}
LVPW;d (mm)	0.79±0.08	1.04±0.13^{A}	0.80±0.11	1.17±0.14^{A}	0.80±0.11	1.19±0.16^{A}	0.81±0.11	1.18±0.14^{A}
EF (%)	65.14±8.59	52.22±5.44^{A}	63.89±14.53	43.87±6.6^{A,B}	50.93±4.52	31.80±6.2^{A,B}	64.19±6.94	45.86±4.2^{A,C}
FS (%)	33.15±4.62	25.67±2.38^{A}	32.72±7.76	21.64±3.4^{A,B}	25.81±2.71	15.76±3.1^{A,B}	32.44±3.69	22.80±2.2^{A,C}

LVID;d, left ventricular internal dimension diastolic; LVPW;d, left ventricular posterior wall diastolic; FS, fractional shortening; EF,ejection fraction. BW, body weight; HW, heart weight; LW, lung weight; TIBIA, tibia length; HW/TIBIA,heart weight/tibia length ratio; HW/BW, heart weight/body weight ratio;LW/TIBIA, lung weight/tibia length ratio.;LV, left ventricle; IVS, interventricular septum. ^{A}, $p<0.05$, TAC vs sham in the same group; ^{B}, $p<0.05$, vs. MerCreMer^{+} TAC; ^{C}, $p<0.05$, vs. Atg5KO TAC;

Table S3. Cardiac remodeling and dysfunction in Ctl, Nrf2KO, CR-Atg5KO and Duo-KO mice at 6 weeks after TAC

	Ctl		Nrf2KO		Atg5KO		Duo-KO	
	Sham	TAC	Sham	TAC	Sham	TAC	Sham	TAC
Echo (n)	(8)	(7)	(10)	(8)	(12)	(6)	(11)	(7)
IVS;d (mm)	1.18±0.12	1.35±0.12[A]	28.65±2.10	1.16±0.12[A,C]	0.92±0.16[B]	1.03±0.11[C]	0.75±0.09[B,D]	1.07±0.10[A]
LVID;d (mm)	3.67±0.44	4.06±0.43[A]	3.72±0.36	3.80±0.32	4.16±0.24[B]	4.93±0.24[A,C]	3.72±0.21[D]	4.00±0.40[E]
LVPW;d (mm)	0.92±0.13	1.40±0.23[A]	0.84±0.07	1.12±0.19[A,C]	0.82±0.14	1.00±0.14[A,C]	0.89±0.09	1.04±0.16[A]
EF (%)	65.78±8.50	39.69±4.79[A]	61.85±8.73	45.63±5.0[A,C]	47.35±8.22[B]	24.7±6.40[A,C]	57.67±5.53[B,]	38.1±8.37[A,E]
FS (%)	35.77±5.97	19.03±2.54[A]	33.34±6.62	22.52±2.7[A,C]	24.11±4.45[B]	11.5±3.17[A,C]	29.8±3.68[B,D]	18.4±4.67[A,E]
Pathology (n)	(8)	(7)	(10)	(8)	(12)	(6)	(11)	(7)
BW (g)	28.95±1.91	28.65±2.10	28.26±1.90	29.06±2.30	29.3±2.35	28.85±1.66	25.4±2.11[B,D]	26.50±2.95
HW (mg)	140.0±15.1	238.57±24[A]	136.0±10.7	197.5±24[A,C]	172.5±20.9[B]	270±20.9[A,C]	120.9±15[B,D]	201.4±16[A,E]
LW (mg)	187.5±22.5	398.6±112[A]	177.0±15.7	346.3±56.6[A]	272.5±64.9[B]	453.3±66.5[A]	165.5±23.4[D]	377.1±53[A,E]
Tibia (mm)	18.05±0.42	17.66±0.52	17.95±0.30	17.76±0.34	18.17±0.34	18.00±0.21	17.89±0.31	17.97±0.29
HW/Tibia (mg/mm)	7.76±0.84	13.51±1.25[A]	7.58±0.67	11.13±1.4[A,C]	9.51±1.26[B]	15.0±1.13[A,C]	6.76±0.89[B,D]	11.21±0.9[A,E]
LW/Tibia (mg/mm)	10.39±1.18	22.61±6.46[A]	9.86±0.90	19.52±3.26[A]	14.98±3.53[B]	25.19±3.73[A]	9.26±1.40[D]	21.01±3.1[A,E]
Remodeling (n)	(4)	(4)	(4)	(4)	(4)	(4)	(4)	(4)
Myocytes (μm^2)	207.1±37.6	403.2±59.8[A]	206.4±43.1	325.2±56[A,B]	286.1±52.7[B]	438.8±65[A,C]	202±31.6	332.6±61[A,E]
Fibrosis (%)	3.40±1.83	23.85±6.72[A]	3.3±1.9	18.9±4.9[A,B]	12.75±4.24[B]	28.45±6.2[A,C]	7.4±3.5[D]	23.1±5.9[A,E]
qPCR (n)	(4)	(4)	(4)	(4)	(4)	(4)	(4)	
α-MHC	1.02±0.18	0.60±0.17[A]	0.98±0.12	0.94±0.22[C]	0.81±0.18[B]	0.37±0.10[A,C]	1.08±0.14[D]	0.63±0.15[A,E]
β-HMC	1.02±0.19	10.16±4.19[A]	1.25±0.18[B]	4.43±1.63[A,C]	11.42±2.35[B]	87.9±12.2[A,C]	2.71±0.52[B,D]	37.2±12.6[A,E]
ANF	1.05±0.30	27.80±2.96[A]	1.03±0.21	14.4±5.6[A,C]	23.46±5.92[B]	47.51±12[A,C]	1.35±0.13[B,D]	19.6±3.56[A,E]
BNP	1.03±0.25	5.42±1.56[A]	1.03±0.24	3.25±0.86[A,C]	4.22±1.30[B]	10.8±1.9[A,C]	2.35±0.54[B,D]	5.85±0.89[A,E]
SERCA2α	1.02±0.20	0.54±0.09[A]	0.98±0.21	0.71±0.15[A,C]	0.65±0.15[B]	0.38±0.09[A,C]	1.06±0.19[D]	0.72±0.23[A,E]

[A], $p<0.05$, TAC vs sham in the same group; [B], $p<0.05$, vs. Ctl sham; [C], $p<0.05$, Ctl TAC; [D], $p<0.05$, vs. Atg5KO sham; [E], $p<0.05$ vs. Atg5KO TAC
